# Survival after spinal surgery for metastases in men with castration-sensitive vs castration-resistant prostate cancer: a nationwide register-based study

**DOI:** 10.1038/s41598-025-34335-2

**Published:** 2026-01-07

**Authors:** Johan Wänman, Mehdy Farhang, Helena Nyström, Johan Styrke, Christel Häggström, Pär Stattin, Sead Crnalic

**Affiliations:** 1 Department of Diagnostics and Intervention, Umeå University, Umeå, Sweden; 2 Department of Surgical Sciences, Uppsala University, Uppsala, Sweden

**Keywords:** Cancer, Oncology, Urology

## Abstract

This nationwide register-based cohort study examined the association between castration status and postoperative survival in men who had undergone surgery for spinal metastases from prostate cancer. Bone metastases are common in prostate cancer, with the spine being the most frequent site. Using data from the Swedish Spine Register (Swespine) and Prostate Cancer Database Sweden (PCBaSe), 306 men with prostate cancer who underwent spinal surgery were identified. In total, 81 were categorized as castration-sensitive and 225 as castration-resistant disease at the time of spinal surgery. Postoperative survival was estimated using Kaplan–Meier analysis and compared with the log-rank test. Multivariable Cox regression was used to adjust for potential confounders. Median survival after surgery was significantly longer in men with castration-sensitive prostate cancer (33 months, IQR 15–55) compared to those with castration-resistant disease (8 months, IQR 5–31; p < 0.001). Castration-sensitive status was independently associated with a lower risk of death (hazard ratio 0.29, 95% CI: 0.20–0.41). These findings indicate that castration sensitivity is a strong prognostic factor for survival after surgery for spinal metastases from prostate cancer and should be considered in surgical decision-making.

**Supplementary Information:**

The online version contains supplementary material available at 10.1038/s41598-025-34335-2.

## Introduction

Bone metastases are found in approximately 10% of men with prostate cancer at the time of initial diagnosis and are found in more than 80% of patients with advanced metastatic disease ^1,2^. Metastasis can occur either during the castration-sensitive stage or after the disease progresses to a castration-resistant state ^3^. The spine is the most common site of bone metastasis ^2^. Patients with spinal metastases often present with pain, pathological fractures, and/or acute progressive neurological symptoms due to metastatic spinal cord compression (MSCC) ^4^.

Compared with radiotherapy alone, surgery combined with radiotherapy has been shown to improve outcomes in patients with MSCC ^4^. However, surgery is associated with high risks of complications, morbidity and mortality ^5,6^, and is generally only recommended if the life expectancy is expected to exceed 3‒6 months ^7^. The process for selecting patients with spinal metastases or MSCC who would most likely benefit from treatment is complex, and the estimation of life expectancy is crucial. However, the factors influencing survival following surgical treatment for spinal metastases of prostate cancer remain poorly understood.

In a systematic review, castration-sensitivity and a high Karnofsky performance status were associated with prolonged survival following surgery for spinal metastases ^8^. Several cohort studies have revealed an association between the castration state at the time of spinal surgery and survival in prostate cancer patients with MSCC. However, the number of patients with castration-sensitive prostate cancer was consistently low across these studies ^9–13^. Furthermore, the castration-sensitive state was not included as a factor in a later meta-analysis on overall survival in patients with spinal metastases of prostate cancer ^14^.

The aim of this study was to investigate the association between castration state and survival after surgery for spinal metastases of prostate cancer in a large national multi-register cohort.

## Materials and methods

### Study design, data sources and setting

This observational study was based on data derived from the Swedish Spine Register (Swespine), the National Prostate Cancer Register (NPCR) of Sweden and the Prostate Cancer Database Sweden (PCBaSe). Swespine has collected data pertaining to spinal surgery since 1993 and data pertaining to surgery for spinal metastases since 2006. Currently, approximately 90% of all spinal surgery units (including all tumor surgery units) in Sweden report to Swespine, and the completeness of patient data is estimated to be approximately 75% ^15^. The aim of NPCR is to provide a basis for analysis of adherence to national guidelines for prostate cancer with the ultimate aim to improve prostate cancer care for all men in Sweden ^16,17^. For this purpose, the NPCR registers data on cancer characteristics at the time of diagnosis, diagnostic workup, and primary treatment. In PCBaSe, data in the NPCR have been linked to other nationwide health care registers, including the Prescribed Drug Register, the Patient Register, and the Cause of Death Register ^18,19^.

### Patient selection

We obtained data pertaining to surgeries for spinal metastases classified as either prostate cancer or unknown primary tumors performed between 2011 and 2021 (*n* = 882) from the Swespine. Only data from the first surgery for each patient was included in the analyses (*n* = 760). Using the unique personal identity numbers assigned to residents in Sweden, these data were linked to the NPCR to verify the prostate cancer diagnosis and then matched with data pertaining to cancer treatment, classification, and survival in the PCBaSe.

**Fig. 1:**
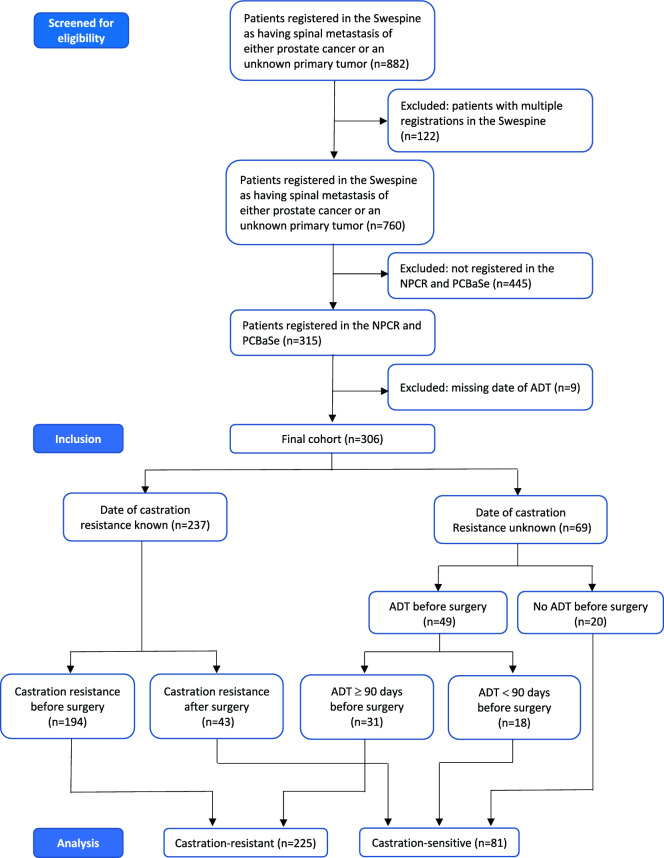
Flowchart of the study.

The quality of the data was controlled by removing data pertaining to other primary tumor diagnoses for patients who were registered as having an unknown primary tumor at the time of spinal surgery. We also evaluated duplicate registrations; only the first registration was used. Furthermore, 9 patients were excluded because of an uncertain castration state. The final cohort included 306 patients registered in all three registers (Fig. 1).

### Definition of castration-sensitive and castration-resistant prostate cancer

Information on the date of castration resistance was available in PCBaSe for 237 men (Fig. 1). They were classified as castration-sensitive at the time of spinal surgery if surgery occurred before the recorded date of castration resistance, and as castration-resistant if surgery occurred after this date. Symptomatic spinal metastasis that led to spinal surgery was the first manifestation of malignancy in 63 patients, whose prostate cancer diagnosis was established only postoperatively. These patients were therefore classified as castration-sensitive at the time of surgery; among them, 43 had available dates of castration resistance, all occurring after surgery.

In addition, 49 patients had received androgen-deprivation therapy (ADT)—orchidectomy, GnRH agonists, or anti-androgens—prior to spinal surgery but lacked recorded information on castration resistance in the registry. For these patients, ADT type and initiation date were available, and castration resistance was therefore assigned using a clinical definition based on radiographic and/or clinical progression 90 days after ADT (Fig. 1). The presence of spinal metastases causing metastatic spinal cord compression or spinal instability requiring surgical intervention was considered as evidence of such progression ^20,21^.

To evaluate the validity of this classification, we conducted sensitivity analyses using two other definitions of castration sensitivity at surgery, (i) spinal surgery before start of ADT (n = 63 castration-sensitive; n = 243 castration-resistant) and (ii) spinal surgery within 180 days after start of ADT (n = 96 castration-sensitive; n = 215 castration-resistant).

### Variables included

We collected information regarding age at prostate cancer diagnosis, PSA at diagnosis, TNM stage, Gleason score, primary treatment and date of death from the NPCR. Data pertaining to age at surgery, date of surgery, type of surgery, and neurological function prior to surgery according to the Frankel classification were collected from the Swespine. Neurological function was further categorized using the Frankel classification, with patients being classified as nonambulatory (Frankel A, B or C) or ambulatory (Frankel D or E). Using data from the PCBaSe, three different comorbidity indices were calculated: the Charlson Comorbidity Index (CCI), the Drug Comorbidity Index (DCI), and the Multi-Dimensional Comorbidity Index (MDCI) ^22–26^. For all three indices, we used the date of spinal surgery as the last date of collection of comorbidity data from prior records in The Patient Register and The Prescribed Drug Register.

### Statistics

Normally distributed variables are presented as the means with standard deviations (SDs), and nonnormally distributed variables are presented as the medians with interquartile ranges (IQRs). Categorical variables were analyzed with the chi-square test. The start of follow**-**up was the date of spinal surgery, and the end of follow**-**up was the date of death or the administrative end of follow**-**up (31^st^ December 2023), whichever came first. Survival is presented in months.

Crude analysis of survival is displayed in Kaplan–Meier figures. Differences between survival curves were assessed with the log-rank test. Cox proportional hazards with adjusted analyses were performed to evaluate the association between castration state and postoperative survival. The covariates included in the multivariate model were castration state, age at spinal surgery, year of surgery, MDCI, and DCI**,** all variables were available for all patients. As, 13 patients had missing data for ambulation, a secondary Cox proportional hazard model was also used to evaluate castration state at the time of spinal surgery after adjusting for ambulation, age at surgery and CCI score. The assumption of proportional hazard was tested with Shoenfeld residuals. The associations are presented as hazard ratios (HRs) with 95% confidence intervals (CIs).

A p value < 0.05 was considered to indicate statistical significance. STATA version 17.0 was used for the statistical analysis.

## Results

### Study patients and descriptive data

The final study cohort comprised 306 men, 81 were classified as having castration-sensitive prostate cancer and 225 classified as having castration-resistant prostate cancer (CRPC) at the time of surgery for spinal metastases (Fig. 1). Among the 81 men with castration-sensitive disease, 63 presented with metastatic spinal cord compression as the initial manifestation of cancer, whereas 18 developed metastases following either curative-intent therapy or during watchful waiting or active surveillance.

The characteristics of the patients at the time of spinal surgery are presented in Table 1 and Table 2, and those at the time of prostate cancer diagnosis are presented in the Supplementary material (Supplementary Table 1). The median age at the time of prostate cancer diagnosis was 67 years (IQR 61–73), and the median age at the time of surgery was 71 years (IQR 67–77). The thoracic spine was the most common location for metastases (n = 191).

**Table 1:** Baseline characteristics of men with spinal metastases of prostate cancer at the time of spinal surgery.

	Castration-resistant^a^ (n = 225)	Castration-sensitive (n = 81)
**Age at surgery, year (median)**	71.0(IQR 67.0–78.0)	70.0(IQR 66.0–76.0)
**Age at surgery**		
< 59 years	16 (7.1%)	7 (8.6%)
60–69	74 (32.9%)	30 (37.0%)
70–79	91 (40.4%)	28 (34.6%)
> 80	44 (19.6%)	16 (19.8%)
**Year of surgery**		
2010–2014	91 (40.4%)	21 (25.9%)
2015–2018	76 (33.8%)	36 (44.4%)
2019–2021	58 (25.8%)	24 (29.6%)
**Time between prostate cancer diagnosis and spinal surgery (median), years**	4.2 (2.0–8.3)	0 (0–0.1)
CCI^b^ score 0	80 (35.6%)	21 (25.9%)
CCI score 1–3	50 (22.2%)	19 (23.5%)
CCI score 4–6	68 (30.2%)	28 (34.6%)
CCI score 7 and above	27 (12.0%)	13 (16.0%)
**Spinal metastases as the first sign of malignancy**		
No	225 (100.0%)	18 (22%)
Yes	0 (0.0%)	63 (78%)
**Frankel**^**c**^** classification**		
A	6 (2.7%)	5 (6.2%)
B	9 (4.0%)	6 (7.4%)
C	99 (44.0%)	39 (48.1%)
D	79 (35.1%)	23 (28.4%)
E	22 (9.8%)	5(6.2%)
Missing	10 (4.4%)	3 (3.7%)

^a^Castration-resistant prostate cancer; Patients who were classified as having castration-resistant disease in PCBaSe at the time of spinal surgery, or who had received androgen deprivation therapy (ADT), i.e

orchidectomy, GnRH agonist or antagonists or anti-androgen more than 3 months before the date of surgery.

for spinal metastases were classified to be in castration-resistant state.

^b^Charlson comorbidity index,

^c^Grade A: complete lesion (paraplegia); grade B: only sensory function; grade C: motor function present but not of practical use (non-ambulatory); grade D: motor function present, sufficient to allow walking (ambulatory); grade E: no neurological symptoms.

**Table 2:** Surgical treatment of the spine.

	Castration-resistant (n = 225)	Castration-sensitive (n = 81)
**Indication for surgery**		
Loss of neurological function	126 (56.0%)	40 (49.4%)
Pain	13 (5.7%)	4 (4.9%)
Progressive deformity	3 (1.3%)	0 (0%)
Loss of neurological function, and pain	59 (26.2%)	28 (34.6%)
Loss of neurological function and progressive deformity	3 (1.3%)	1 (1.2%)
Pain and progressive deformity	5 (2.2%)	1 (1.2%)
Loss of neurological function, pain and progressive deformity	14 (6.2%)	7 (8.6%)
Missing	2 (0.9%)	0 (0%)
**Level of surgery**		
Cervical	10 (4.4%)	5 (6.2%)
Thoracic	140 (62.2%)	51 (63.0%)
Lumbar	47 (20.9%)	13 (16.0%)
Missing	28 (12.4%)	12 (14.8%)
**Surgical decompression**		
Yes	224 (99.6%)	81 (100%)
No	1 (0.4%)	0 (0%)
**Stabilization**		
Yes	134 (59.6%)	61 (75.3%)
No	84 (37.3%)	18 (22.3%)
Missing	7 (3.1%)	2 (2.5%)
**Tumour resection**		
Yes	182 (80.9%)	62 (76.5%)
No	36 (16%)	14 (17.3%)
Missing	7 (3.1%)	5 (6.2%)
**Type of tumour resection**		
Wide surgical excision	19 (8.4%)	4 (4.9%)
Marginal excision	30 (13.3%)	7 (8.6%)
intralesional excision	134 (59.6%)	51 (63%)
RF ablation	1 (0.4%)	0 (0%)
missing	41 (18.2%)	19 (23.4%)

### Neurological function prior to surgery

Before surgery, 129 of the patients were ambulatory (Frankel grades D and E), 164 patients had lost their ability to walk (Frankel grades A, B, and C), and ambulatory function data were missing for 13 patients (Table 1).

### Treatment

#### Primary treatment at the time of prostate cancer diagnosis

Radical prostatectomy was performed in 36 patients, external curative radiotherapy in 18 patients, brachytherapy in 3 patients, external therapy in combination with brachytherapy in 6 patients, and undefined-type radiotherapy in 2 patients. Noncurative radiotherapy was performed in 212 patients, and active surveillance or watchful waiting was initiated in 17 patients. Three patients had died before the decision was made about primary treatment, and 9 had missing information pertaining to primary treatment. The most common hormone therapy was a combination of GnRH and antiandrogens for flare-up; 267 patients received GnRH, 21 underwent orchidectomy, and 270 patients received antiandrogens either before or after spinal surgery (Supplementary Table 2).

#### Surgical treatment of the spine

In the total cohort (n = 306), loss of neurological function was the most common indication for surgery, reported in 166 patients. Pain alone was the primary indication in 17 patients, and progressive deformity alone in 3 patients. Combined indications were frequent: 87 patients presented with both neurological impairment and pain, 4 with neurological impairment and progressive deformity, and 6 with pain and progressive deformity. Additionally, 21 patients presented with all three indications, neurological impairment, pain, and progressive deformity. Indication data were missing for 2 patients. Most patients underwent decompression surgery (n = 305). Stabilization was performed in 195 patients and data on stabilization data were unavailable for 9 patients (Table 2). Radiotherapy to the spinal lesion was administered prior to spinal surgery in 10 patients, with a median interval of 15 months (IQR 4–29). Postoperative radiotherapy to the spine was given to 27 patients, with a median interval of 1 month after surgery (IQR 0.5–7).

#### Survival

**Fig. 2:**
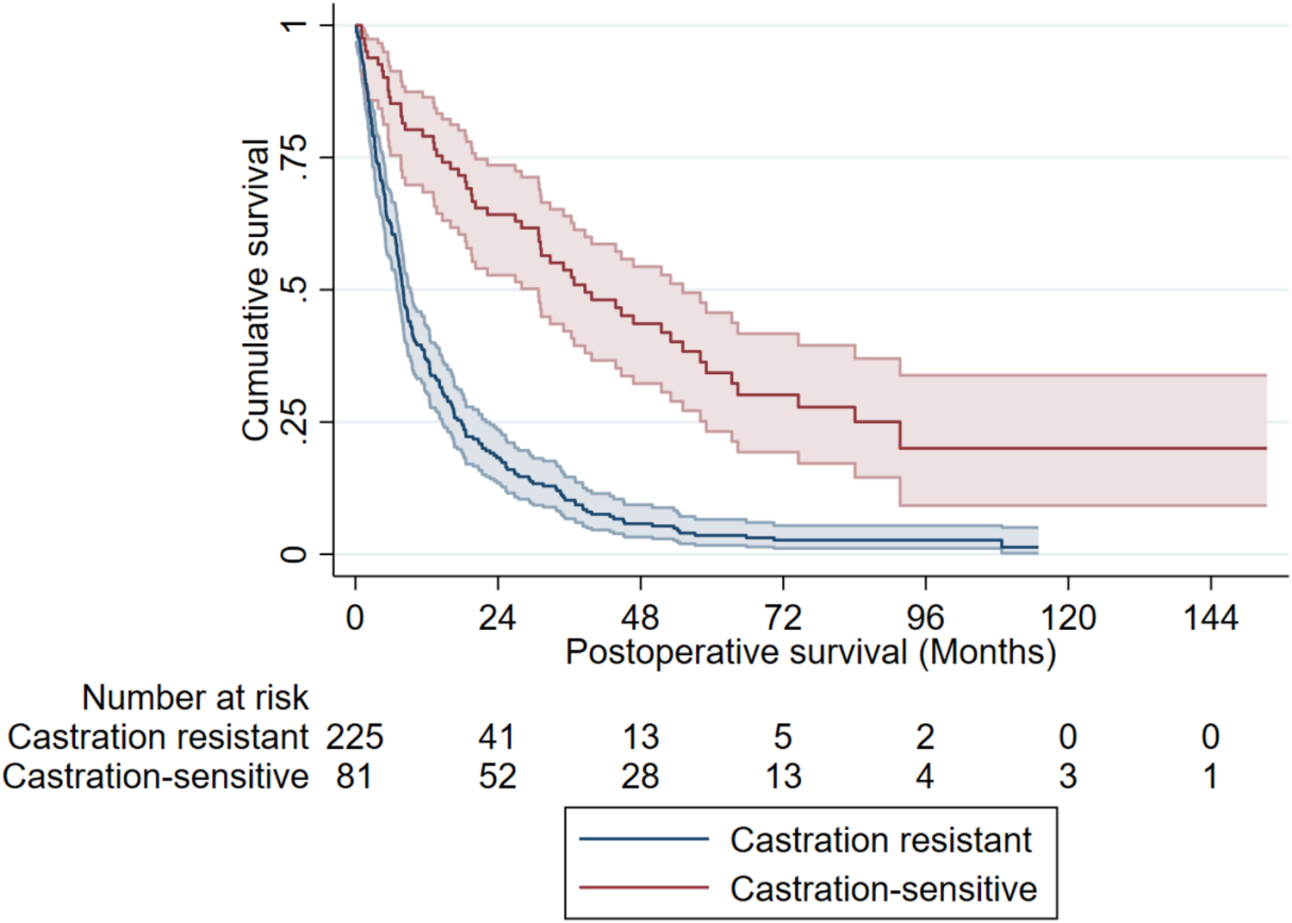
Kaplan‒Meier postoperative survival curves. Postoperative survival was significantly longer in patients with castration-sensitive prostate cancer, 33 (IQR 15–55) months, than in patients with castration-resistant prostate cancer (CRPC), 8 (IQR 4–18) months, (p < 0.001).

The median postoperative survival for the entire cohort was 11 months (IQR 5–31). Survival was significantly longer in the group with castration-sensitive disease, who had a median survival of 33 months (IQR 15–55), compared with 8 months (IQR 4–18) in the CRPC group (p < 0.001) (Fig. 2). In the multivariable model, castration-sensitive disease remained independently associated with longer survival after surgery (HR 0.29, 95% CI: 0.20–0.41) compared with castration-resistant disease (Table 3, Supplementary Table 2). The findings were consistent across the main analysis and both complementary sensitivity analyses, with castration state at the time of surgery persistently showing strong association with survival (Supplementary Table 3). Ambulatory status was not associated with postoperative survival (Supplementary Table 2). Patients who were ambulatory prior to surgery had a median postoperative survival of 11 months (IQR 5–30), whereas non-ambulatory patients had a median postoperative survival of 10 months (IQR 4–30), (p = 0.75).

**Table 3:** Variables associated with the risk of death after surgery for spinal metastases of prostate cancer.

	HR^a^	95% CI	p value
Castration-resistant	Ref		
Castration-sensitive	0.36	0.26–0.50	< 0.001
Age at surgery	1.02	1.0–1.0	0.002
DCI^b^	1.09	1.0–1.2	0.002
MDCI^c^	1.28	1.1–1.5	0.005
2010–2014	Ref		
2015–2018	0.77	0.6–1.0	0.064
2019–2021	1.09	0.8–1.5	0.593

^a^Hazard Ratio.

^b^Drug comorbidity index.

^c^Multidimensional comorbidity index.

## Discussion

In this nationwide register-based study of spinal metastases from prostate cancer, men with castration-sensitive disease had fourfold longer survival after spinal surgery than men with CRPC.

The presence of spinal metastases typically indicates a poor prognosis, making careful patient selection a critical task. This selection process is based on life expectancy, among other factors. Advances in oncology have significantly improved the prognosis for certain tumors, although these improvements can complicate accurate survival estimation.

Our results are in accordance with previous reports on the impact of castration state on survival in prostate cancer patients with spinal metastases ^8–13^. These studies included few patients with castration-sensitive tumors and a short postoperative follow-up. Huddart et al. ^13^ reported that among 17 patients, 13 underwent surgical treatment, and 4 received radiotherapy, with a median survival of 627 days after treatment. In a study by Crnalic et al. ^10^, 8 out of 13 castration-sensitive patients were still alive after surgery, with a median postoperative follow-up of 26 months. Wänman et al. ^9^ reported a median postoperative survival of 60 months for patients in the castration-sensitive group (n = 26) compared with 5.5 months for patients with CRPC (n = 84) in a local cohort of 110 prostate cancer patients. In a study by Ju et al. ^12^, all 3 castration-sensitive patients were alive at 37 months, 40 months, and 46 months after surgery. A recent systematic review and meta-analysis assessed prognostic factors for survival in patients with spinal metastases secondary to prostate cancer ^14^. Factors such as visceral metastases, ambulatory status, extraspinal bone metastases, performance status, time to motor deficit development, and time from primary diagnosis were identified as significantly associated with overall survival ^14^. However, the castration state was not included in the analysis, likely due to the limited number of patients with castration-sensitive prostate cancer.

In this national cohort, postoperative survival was four times longer in the castration-sensitive group than in the CRPC group. The median survival of 33 months for castration-sensitive prostate cancer patients in our study was comparable to the survival reported for castration-sensitive prostate cancer patients with bone metastases in general ^27^. This contrasts with the typically reported median survival range of 6–9 months for patients with spinal metastases ^28,29^, which aligns with the survival observed in the CRPC group in our study.

Our findings highlight the large range in prognosis among men with spinal metastases from prostate cancer. Although survival differences between castration-sensitive and castration-resistant disease are well recognized, our study adds context-specific evidence by demonstrating how castration status at the time of surgery is associated with postoperative survival in patients undergoing spinal procedures. This information can support clinical decision-making by refining expectations regarding outcomes and reinforcing the need for a tailored, multidisciplinary treatment strategy, including awareness of therapeutic options and realistic postoperative survival estimation when considering surgery.

Our classification of castration-sensitive and castration-resistant disease relied on registry data and was supplemented, when necessary, by clinical criteria based on ADT timing and documented progression to spinal metastases. The consistency of results in sensitivity analyses using alternative timeline definitions strengthens the validity of our classification.

We used the CCI, DCI and MDCI to evaluate comorbidities. All three indices were associated with postoperative survival. Similarly, these indices have been validated in previous studies on prostate cancer, confirming their value as tools for assessing comorbidities and their reliability in evaluating survival outcomes in prostate cancer patients ^25,26^. While a previous study revealed an association between preoperative ambulation ability and improved postoperative survival ^28^, our findings did not confirm this relationship.

Nater et al. ^29^ highlighted that the quality of evidence for current predictors of survival following surgery for symptomatic spinal metastases is low. Prostate cancer bone metastases are highly heterogeneous at the genomic, transcriptomic, proteomic, metabolomic and morphological levels, all of which significantly influence survival outcomes ^30^. A deeper understanding of the complex biology of metastatic prostate cancer, particularly distinguishing castration-sensitive disease as a potentially distinct entity with significant prognostic implications, may enhance the selection of surgical candidates and lead to improved treatment outcomes.

### Strengths and limitations

The main strengths of this study include its nationwide design, which provides strong external validity and generalizability, and its large sample size, which ensures high statistical power. Additionally, the depth of information available from high-quality national registries enhances the robustness of the study. The personal identification numbers assigned to all residents of Sweden also facilitate long-term follow-up on survival. The comorbidity indices were adjusted in this study to provide a comparable risk of death, which is in line with the results of randomized control trials ^31^.

The main limitation is the retrospective register-based design of the study, which has inherent limitations, including miscoding, transferring errors, underreporting, and missing data ^15^. However, the data in registries were prospectively collected, minimizing the risk of recall bias and we argue that it is unlikely that these limitations affected men with castration sensitive and CRPC differently. Another limitation is that only patients selected for surgery were included, which introduces the potential for selection bias, as these patients are likely to have a more favorable prognosis. A further limitation is the limited information on systemic therapies in the national registers. Although castration status was available, detailed data on specific treatments including second-generation androgen receptor inhibitors, chemotherapy, radiopharmaceuticals, and bone-protecting agents were not captured. Similarly, important clinical parameters such as bone marrow involvement, baseline hemoglobin, platelet count, and LDH were not systematically recorded, limiting our ability to account for cytopenias or bone marrow infiltration, both of which have prognostic relevance in metastatic prostate cancer ^32^. Additionally, the lack of detailed data on metastatic burden, including metastatic volume, presence of visceral metastases, and the number of affected vertebral levels, were not consistently available in the registers. To partially address patient heterogeneity, our analyses adjusted for comorbidity burden and neurological function at presentation, using MDCI, CCI, and ambulation/Frankel grade.

## Conclusion

In this large cohort of men with spinal metastases from prostate cancer, men who underwent spinal metastasis surgery with castration sensitive cancer, had a four-fold longer survival than men with CRPC. Castration state should be considered when selecting prostate cancer patients for spinal surgery.

## Supplementary Information

Supplementary Table 1.

Supplementary Table 2.

Supplementary Table 3.

## Data Availability

These data cannot be shared publicly because the individual-level data contain potentially identifying and sensitive patient information and cannot be published owing to legislation and ethical review restrictions ( [https://etikprovningsmyndigheten.se](https:/etikprovningsmyndigheten.se) ). The use of data from national health data registers is further restricted by the Swedish Board of Health and Welfare ( [https://www.socialstyrelsen.se/en/](https:/www.socialstyrelsen.se/en) ) and Statistics Sweden ( [https://www.scb.se/en/](https:/www.scb.se/en) ), which are government agencies providing access to linked healthcare registers.
