# Supplementary material for: Survival after spinal surgery for metastases in men with castration-sensitive vs castration-resistant prostate cancer: a nationwide register-based study
Source: Sci Rep. 2026 Jan 7;16:887. doi: 10.1038/s41598-025-34335-2 (PMC12783619; doi:10.1038/s41598-025-34335-2)
Supplement: Supplementary file 1 — Supplementary Table 1. [file 41598_2025_34335_MOESM1_ESM.docx]

**Supplementary Table 1. Clinical characteristic and primary treatment at the time of diagnosis of prostate cancer**

|  | Castration-resistant ^a^ | Castration sensitive | Total |
| --- | --- | --- | --- |
| **Age at diagnosis (median, IQR)** | 66 (60-71) | 69 (65-75) | 67 (61-73) |
| **PSA^b^ at diagnosis (median, IQR)** | 37 (13-225) | 284 (37-894) | 130 (31-652) |
| **TNM^c^** |  |  |  |
| **T** |  |  |  |
| T0 | 1 | 2 | 3 |
| T1 | 30 | 14 | 44 |
| T2 | 64 | 18 | 82 |
| T3 | 98 | 21 | 119 |
| T4 | 3 | 14 | 17 |
| Not possible to judge | 3 | 14 | 17 |
| Missing | 0 | 2 | 2 |
| **N** |  |  |  |
| N0 | 34 | 17 | 51 |
| N1 | 42 | 11 | 53 |
| Not possible to judge | 148 | 53 | 201 |
| Missing | 1 | 0 | 1 |
| **M0** | 78 | 18 | 100 |
| M1 | 99 | 63 | 153 |
| Missing | 48 | 0 | 53 |
| **Gleason score** |  |  |  |
| 10 | 3 | 0 | 3 |
| 9 | 68 | 21 | 89 |
| 8 | 47 | 3 | 50 |
| 7 | 65 | 12 | 77 |
| 6 | 21 | 8 | 29 |
| 5 | 5 | 3 | 8 |
| Missing | 16 | 34 | 50 |
| **Primary treatment** |  |  |  |
| Radiotherapy | 41 | 3 | 44 |
| Radical Prostatectomy | 16 | 5 | 21 |
| Active surveillance | 2 | 7 | 9 |
| Conservative: type missing | 5 | 3 | 8 |
| Non-curative radiotherapy | 152 | 60 | 212 |
| Dead before treatment decision | 0 | 3 | 3 |
| Missing information about Radiotherapy | 9 | 0 | 9 |
| **Hormone therapy** |  |  |  |
| Orchiectomy | 6 | 5 | 11 |
| Orchiectomy +antiandrogens | 2 | 3 | 5 |
| GnRH^d^ | 28 | 17 | 45 |
| GnRH+ antiandrogens flair-protection | 71 | 23 | 94 |
| GnRH + antiandrogens continuously | 19 | 3 | 22 |
| GnRH+ antiandrogens duration unknown | 2 | 4 | 6 |
| Antiandrogens flare protection | 2 | 1 | 3 |
| Antiandrogens continuously | 19 | 2 | 21 |
| Antiandrogens duration missing | 2 | 2 | 4 |

^a^Castration-resistant prostate cancer; Patients who had received androgen deprivation therapy (ADT), i.e.

orchidectomy, GnRH agonist or antagonists or anti-androgen more than 3 months before the date of surgery

for spinal metastases were classified to be in castration-resistant state.

^b^Prostate-Specific Antigen

^c^Tumor, Nodes, Metastases (TNM)-Classification.

^d^ Gonadotropin-releasing hormone.
