# Supplementary material for: Survival after spinal surgery for metastases in men with castration-sensitive vs castration-resistant prostate cancer: a nationwide register-based study
Source: Sci Rep. 2026 Jan 7;16:887. doi: 10.1038/s41598-025-34335-2 (PMC12783619; doi:10.1038/s41598-025-34335-2)
Supplement: Supplementary file 2 — Supplementary Table 2. [file 41598_2025_34335_MOESM2_ESM.docx]

**Supplementary Table 2. Variables associated with the risk of death after surgery for spinal metastases of prostate cancer**

|  | HR^a^ | 95% CI | P-value |
| --- | --- | --- | --- |
| Castration resistant | Ref |  |  |
| Castration sensitive | 0.28 | 0.21-0.39 | <0.001 |
| Age at surgery | 1.02 | 1.0-1.0 | 0.032 |
| CCI^b^ 0 | Ref | . | . |
| CCI 1-3 | 1.06 | 0.76-1.47 | 0.73 |
| CCI 4-6 | 0.88 | 0.64-1.20 | 0.41 |
| CCI 7 and above | 0.96 | 0.64-1.44 | 0.85 |
| Non-ambulatory | Ref | . | . |
| Ambulatory | 0.83 | 0.64-1.07 | 0.16 |

^a^ Hazard Ratio

^b^Charlsson comorbidity index.
