# Supplementary material for: Survival after spinal surgery for metastases in men with castration-sensitive vs castration-resistant prostate cancer: a nationwide register-based study
Source: Sci Rep. 2026 Jan 7;16:887. doi: 10.1038/s41598-025-34335-2 (PMC12783619; doi:10.1038/s41598-025-34335-2)
Supplement: Supplementary file 3 — Supplementary Table 3. [file 41598_2025_34335_MOESM3_ESM.docx]

**Supplementary table 3. Variables associated with the risk of death after surgery for spinal metastases of prostate cancer.**

A. Model 2: Initial manifestation of malignancy with no ADT prior to surgery categorized as castration-sensitive

|  | HR^a^ | 95% CI | p value |
| --- | --- | --- | --- |
| Castration-resistant | Ref |  |  |
| Castration-sensitive | 0.39 | 0.27-0.55 | <0.001 |
| Age at surgery | 1.02 | 1.0-1.0 | 0.009 |
| DCI^b^ | 1.10 | 1.1-1.2 | <0.001 |
| MDCI^c^ | 1.29 | 1.0-1.5 | 0.017 |
| 2010-2014 | Ref |  |  |
| 2015-2018 | 0.76 | 0.6-1.0 | 0.060 |
| 2019-2021 | 1.06 | 0.8-1.5 | 0.703 |

**B. Model 3: ADT less then 180 days before surgery** categorized as castration-sensitive

|  | HR^a^ | 95% CI | p value |
| --- | --- | --- | --- |
| Castration-resistant | Ref |  |  |
| Castration-sensitive | 0.44 | 0.36-0.60 | <0.001 |
| Age at surgery | 1.02 | 1.0-1.0 | <0.001 |
| DCI^b^ | 1.10 | 1.0-1.2 | <0.001 |
| MDCI^c^ | 1.29 | 1.1-1.5 | <0.001 |
| 2010-2014 | Ref |  |  |
| 2015-2018 | 0.78 | 0.6-1.0 | 0.082 |
| 2019-2021 | 1.06 | 0.7-1.5 | 0.704 |

^a^Hazard Ratio

^b^Drug comorbidity index

^c^Multidimensional comorbidity index.

1. Model 2: Spinal surgery before any ADT initiation (n = 63 castration-sensitive; n=243 castration-resistant)
2. Model 3. Spinal surgery within 180 days after first ADT (n = 96 castration-sensitive; n= 215 castration-resistant)
